# The complete chloroplast genome of *Rhododendron kawakamii* (Ericaceae)

**DOI:** 10.1080/23802359.2021.1959439

**Published:** 2021-08-02

**Authors:** Zheng-Feng Wang, Li-Wan Chang, Hong-Lin Cao

**Affiliations:** a Key Laboratory of Vegetation Restoration and Management of Degraded Ecosystems, South China Botanical Garden, Chinese Academy of Sciences, Guangzhou; b Center for Plant Ecology, Core Botanical Gardens, Chinese Academy of Sciences, Guangzhou; c Southern Marine Science and Engineering Guangdong Laboratory (Guangzhou), Guangzhou; d Forest Protection Division, Taiwan Forestry Research Institute, Taipei

**Keywords:** *Rhododendron kawakamii*, chloroplast, genome assembly, next generation sequencing

## Abstract

*Rhododendron kawakamii* is endemic in Taiwan island and is a unique and epiphytic species. Here, we report its complete chloroplast genome. The length of the *R. kawakamii* chloroplast genome is 230,777 bp, with a large single-copy region of 146,155 bp, a small single-copy region of 72,082 bp, and a pair of inverted repeat regions (IRA) of 6,270 bp each. The genome contains 77 protein-coding genes, 29 transfer RNA genes, and four ribosomal RNA genes. In addition, the genome contains 81 simple sequence repeats. Phylogenetic analysis revealed that *R. kawakamii* is genetically related to *R. datiandingense*.

*Rhododendron*, in the Ericaceae family, is a large and extremely diverse genus, containing more than 1000 species worldwide (Shrestha et al. 2018). *Rhododendron kawakamii* Hayata is endemic to Taiwan island. Unlike the other *Rhododendron* species on the island, which are either shrubs or trees, *R*. *kawakamii* is epiphytic (Tsai et al. 2012) and naturally distributed in the forest between 1500 and 2600 m. Molecular data supported its uniqueness in the phylogeny and showed that it formed an independent cluster in section *Vireya* of subgenus *Rhododendron* (Tsai et al. 2003, 2012). Both natural and artificial hybridization among *Rhododendron* species are common due to their low reproduction barriers (Kaul et al. 1986; Milne et al. 2010), which is particularly true in section *Vireya,* possibly as a result of adaptive radiation events (Milne et al. 2010). Crossing experiments indicate that *R*. *kawakamii* could also hybridize with *R*. *santapaui* and produce viable seeds (Kaul et al. 1986). As approximately 70% of *Rhododendron* are classified as endangered or need conservation (Shrestha et al. 2018), and hybridization presents a grave threat to species integrity (Zhang, Qin, et al. 2020b), we report the complete chloroplast genome of *R*. *kawakamii* to provide a germplasm resource for its evolution and conservation studies.

Fresh leaves of *R. kawakamii* were collected from the Shanlinxi forest recreation area (N23°38′7.8″, E120°47′29.3″). A voucher specimen was deposited at the Herbarium of Taiwan Forest Research Institute (https://taif.tfri.gov.tw/tw/index.php, Chien-Fan Chen, chenc@tfri.gov.tw) under the voucher number 511025. The genomic DNA of *R. kawakamii* was extracted by the CTAB (cetyltrimethylammonium bromide) method. The extracted DNA was sequenced using the Illumina HiSeq X Ten system. The sequences were then used to assemble the chloroplast genome of *R. kawakamii* by Fast-Plast 1.2.8 (McKain and Wilson 2017). After assembly, the genome was polished by Pilon 1.24 (Walker et al. 2014) twice. The polished genome was subsequently annotated with CPGAVAS2 (Shi et al. 2019), GeSeq (Tillich et al. 2017), and PGA (Qu et al. 2019). The annotated genome is now available in GenBank under the accession number MW762686. Phylogenetic analysis was perform using maximum likelihood in PhyloSuite 1.2.2 (Zhang, Gao, et al. 2020a) with the concatenated protein sequences of 77 chloroplast coding genes for *R. kawakamii* and the other 17 species.

The *R. kawakamii* chloroplast genome was 230,777 bp in length with a GC content of 35.10%. The genome showed a large single-copy region of 146,155 bp, a small single-copy region of 72,082 bp, and two copies of inverted repeat regions of 6,270 bp each. After annotation, a total of 110 genes were identified in the *R. kawakamii* chloroplast genome, including 77 protein-coding genes, 29 transfer RNA genes, and four ribosomal RNA genes. Furthermore, a total of 81 simple sequence repeats (SSR) were discovered in the genome. These SSRs included 76 mononucleotides (A/T), 3 dinucleotides (TA) and 2 trinucleotides (AAT/ATT). Phylogenetic analysis revealed that *R. kawakamii* was genetically related to *R. datiandingense* (Figure 1).

**Figure 1.**
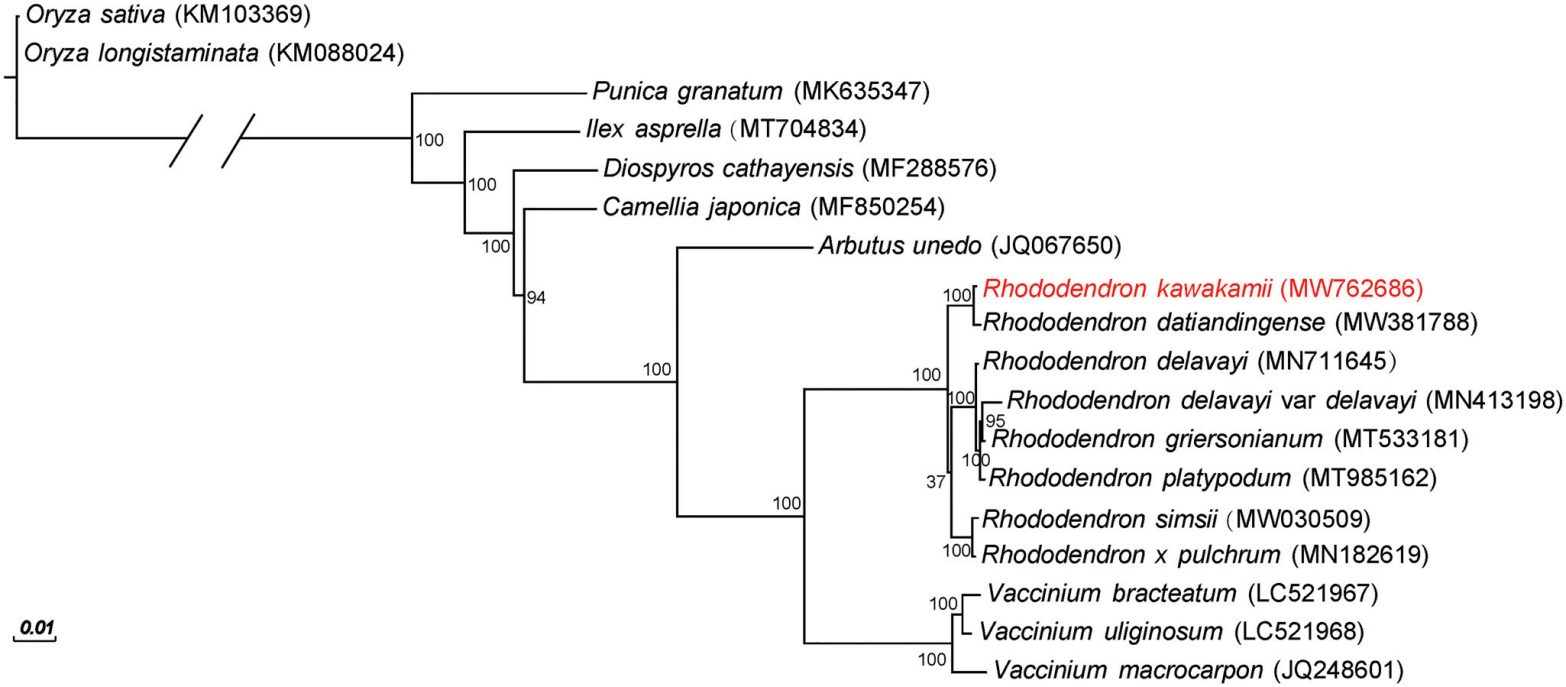
Phylogenetic tree for *Rhododendron kawakamii* and 17 additional species. The GenBank accession numbers are shown in parentheses. Bootstrap values are shown at nodes.

For the 18 species analyzed in phylogeny, a total of 90 protein-coding genes were annotated in their chloroplast genomes (Table S1), in which *clpP*, *lhbA*, *orf23*, *orf28*, *orf34*, *orf39*, *orf46*, *ORF63*, *orf64*, *orf222*, *ycf1*, *ycf2,* and *ycf68* genes were not identified in *R. kawakamii* chloroplast genome. The *orf23*, *orf28*, *orf34*, *orf39*, *orf46*, *ORF63*, *orf64,* and *orf222* genes were only annotated in two *Oryza* species, and their functions were unknown. For *clpP*, *lhbA*, *ycf1*, *ycf2* and *ycf68* genes, they also extensively missed in the other *Rhododendron* species. *clpP* gene is a proteolytic subunit of the ATP-dependent *Clp* protease (Shikanai et al. 2001), may be related to the development of plant (Shikanai et al. 2001; Moreno et al. 2017). *lhbA* encodes a structural protein of the light-harvesting antenna, may be related to maintenance of stable antenna complexes (Ruf et al. 2000). *ycf1*, *ycf2* and *ycf68* genes are in *ycf* gene family and their functions generally unknown (Logacheva et al. 2017; Ślipiko et al. 2020), although *ycf*2 gene is a putative ATPase (Huang et al. 2017). How these missed genes in *R. kawakamii* chloroplast are functionally related to the growth of *R. kawakamii* needs further study in the future.

## Data Availability

The genome sequence data that support the finding of this study are openly available in GenBank of NCBI at [https://www.ncbi.nlm.nih.gov] (https://www.ncbi.nlm.nih.gov/) under the accession number MW762686 and is also accessible at https://doi.org/10.13140/RG.2.2.36349.69600. The associated BioProject, SRA, and Bio-Sample numbers for reads are PRJNA701862, SRR13987606, and SAMN17915251 respectively.

## References

[CIT0001] Huang Y, Wang J, Yang Y, Fan C, Chen J. 2017. Phylogenomic analysis and dynamic evolution of chloroplast genomes in Salicaceae. Front Plant Sci. 8:1050. 28676809 10.3389/fpls.2017.01050 PMC5476734

[CIT0002] Kaul V, Rouse JL, Williams EG. 1986. Early events in the embryo sac after intraspecific and interspecific pollinations in *Rhododendron kawakamii* and *R*. *retusum*. Can J Bot. 64(2):282–291.

[CIT0003] Logacheva MD, Krinitsina AA, Belenikin MS, Khafizov K, Konorov EA, Kuptsov SV, Speranskaya AS. 2017. Comparative analysis of inverted repeats of polypod fern (Polypodiales) plastomes reveals two hypervariable regions. BMC Plant Biol. 17(Suppl 2):255. 29297348 10.1186/s12870-017-1195-z PMC5751766

[CIT0004] McKain M, Wilson M. 2017. Fast-Plast: Rapid de novo assembly and finishing for whole chloroplast genomes. Available form: https://github.com/mrmckain/Fast-Plast.

[CIT0005] Milne RI, Davies C, Prickett R, Inns LH, Chamberlain DF. 2010. Phylogeny of *Rhododendron* subgenus *Hymenanthes* based on chloroplast DNA markers: between-lineage hybridization during adaptive radiation? Plant Syst Evol. 285(3-4):233–244.

[CIT0006] Moreno JC, Tiller N, Diez M, Karcher D, Tillich M, Schöttler MA, Bock R. 2017. Generation and characterization of a collection of knock-down lines for the chloroplast Clp protease complex in tobacco. J Exp Bot. 68(9):2199–2218. 28369470 10.1093/jxb/erx066 PMC5447895

[CIT0007] Qu XJ, Moore MJ, Li DZ, Yi TS. 2019. PGA: a software package for rapid, accurate, and flexible batch annotation of plastomes. Plant Methods. 15:50. 31139240 10.1186/s13007-019-0435-7 PMC6528300

[CIT0008] Ruf S, Biehler K, Bock R. 2000. A small chloroplast-encoded protein as a novel architectural component of the light-harvesting antenna. J Cell Biol. 149(2):369–378. 10769029 10.1083/jcb.149.2.369 PMC2175164

[CIT0009] Shi L, Chen H, Jiang M, Wang L, Wu X, Huang L, Liu C. 2019. CPGAVAS2, an integrated plastome sequence annotator and analyzer. Nucleic Acids Res. 47(W1):W65–W73. 31066451 10.1093/nar/gkz345 PMC6602467

[CIT0010] Shikanai T, Shimizu K, Ueda K, Nishimura Y, Kuroiwa T, Hashimoto T. 2001. The chloroplast clpP gene, encoding a proteolytic subunit of ATP-dependent protease, is indispensable for chloroplast development in tobacco. Plant Cell Physiol. 42(3):264–273. 11266577 10.1093/pcp/pce031

[CIT0011] Shrestha N, Wang Z, Su X, Xu X, Lyu L, Liu Y, Dimitrov D, Kennedy JD, Wang Q, Tang Z, et al. 2018. Global patterns of *Rhododendron* diversity: the role of evolutionary time and diversification rates. Global Ecol Biogeogr. 27(8):913–924.

[CIT0012] Ślipiko M, Myszczyński K, Buczkowska K, Bączkiewicz A, Szczecińska M, Sawicki J. 2020. Molecular delimitation of European leafy liverworts of the genus *Calypogeia* based on plastid super-barcodes. BMC Plant Biol. 20(1):243. 32466772 10.1186/s12870-020-02435-y PMC7257191

[CIT0013] Tillich M, Lehwark P, Pellizzer T, Ulbricht-Jones ES, Fischer A, Bock R, Greiner S. 2017. GeSeq - versatile and accurate annotation of organelle genomes. Nucleic Acids Res. 45(W1):W6–W11. 28486635 10.1093/nar/gkx391 PMC5570176

[CIT0014] Tsai CC, Chen CH, Chou CH. 2012. DNA barcodes reveal high levels of morphological plasticity among *Rhododendron* species (Ericaceae) in Taiwan. Biochem Syst Ecol. 40:169–177.

[CIT0015] Tsai CC, Huang SC, Chen CH, Tseng YH, Huang PL, Tsai SH, Hou CH. 2003. Genetic relationships of *Rhododendron* (Ericaceae) in Taiwan based on the sequence of the internal transcribed spacer of ribosomal DNA. J Hortic Sci Biotechnol. 78 (2):234–240.

[CIT0016] Walker BJ, Abeel T, Shea T, Priest M, Abouelliel A, Sakthikumar S, Cuomo CA, Zeng Q, Wortman J, Young SK, et al. 2014. Pilon: an integrated tool for comprehensive microbial variant detection and genome assembly improvement. PLoS One. 9(11):e112963. 25409509 10.1371/journal.pone.0112963 PMC4237348

[CIT0017] Zhang D, Gao F, Jakovlić I, Zou H, Zhang J, Li WX, Wang GT. 2020a. PhyloSuite: an integrated and scalable desktop platform for streamlined molecular sequence data management and evolutionary phylogenetics studies. Mol Ecol Resour. 20(1):348–355. 31599058 10.1111/1755-0998.13096

[CIT0018] Zhang X, Qin H, Xie W, Ma Y, Sun W. 2020b. Comparative population genetic analyses suggest hybrid origin of *Rhododendron pubicostatum*, an endangered plant species with extremely small populations endemic to Yunnan. Plant Divers. 42(4):312–318. 33094202 10.1016/j.pld.2020.06.012 PMC7567756

